# Enhanced HoVerNet Optimization for Precise Nuclei Segmentation in Diffuse Large B-Cell Lymphoma

**DOI:** 10.3390/diagnostics15151958

**Published:** 2025-08-04

**Authors:** Gei Ki Tang, Chee Chin Lim, Faezahtul Arbaeyah Hussain, Qi Wei Oung, Aidy Irman Yajid, Sumayyah Mohammad Azmi, Yen Fook Chong

**Affiliations:** 1 Faculty of Electronic Engineering and Technology, Universiti Malaysia Perlis, Arau 02600, Perlis, Malaysia; geiki@studentmail.unimap.edu.my (G.K.T.); qiwei@unimap.edu.my (Q.W.O.); 2 Sport Engineering Research Centre, Universiti Malaysia Perlis, Arau 02600, Perlis, Malaysia; fook1987@gmail.com; 3 Department of Pathology, Hospital Universiti Sains Malaysia, Kubang Kerian 16150, Kelantan, Malaysia; faezahtul@usm.my (F.A.H.); aidy@usm.my (A.I.Y.); sumayyah@student.usm.my (S.M.A.); 4 Department of Pathology, School of Medical Sciences, Universiti Sains Malaysia, Kubang Kerian 16150, Kelantan, Malaysia; 5 Communication Engineering (ACE) Centre of Excellence, Universiti Malaysia Perlis, Arau 02600, Perlis, Malaysia

**Keywords:** deep learning, diffuse large B-Cell lymphoma, graphic user interface, HoVerNet, nuclei classification, nuclei segmentation

## Abstract

**Background/Objectives**: Diffuse Large B-Cell Lymphoma (DLBCL) is the most common subtype of non-Hodgkin lymphoma and demands precise segmentation and classification of nuclei for effective diagnosis and disease severity assessment. This study aims to evaluate the performance of HoVerNet, a deep learning model, for nuclei segmentation and classification in CMYC-stained whole slide images and to assess its integration into a user-friendly diagnostic tool. **Methods**: A dataset of 122 CMYC-stained whole slide images (WSIs) was used. Pre-processing steps, including stain normalization and patch extraction, were applied to improve input consistency. HoVerNet, a multi-branch neural network, was used for both nuclei segmentation and classification, particularly focusing on its ability to manage overlapping nuclei and complex morphological variations. Model performance was validated using metrics such as accuracy, precision, recall, and F1 score. Additionally, a graphic user interface (GUI) was developed to incorporate automated segmentation, cell counting, and severity assessment functionalities. **Results**: HoVerNet achieved a validation accuracy of 82.5%, with a precision of 85.3%, recall of 82.6%, and an F1 score of 83.9%. The model showed powerful performance in differentiating overlapping and morphologically complex nuclei. The developed GUI enabled real-time visualization and diagnostic support, enhancing the efficiency and usability of DLBCL histopathological analysis. **Conclusions**: HoVerNet, combined with an integrated GUI, presents a promising approach for streamlining DLBCL diagnostics through accurate segmentation and real-time visualization. Future work will focus on incorporating Vision Transformers and additional staining protocols to improve generalizability and clinical utility.

## 1. Introduction

Accurate segmentation and classification of nuclei are crucial in the analysis of Diffuse Large B-Cell Lymphoma (DLBCL) tissue images [1]. Proper identification and quantification of tumor cells can significantly impact diagnosis and treatment planning. Traditional methods of nuclei segmentation and classification often struggle with challenges such as tissue heterogeneity, staining variability, and complex cellular interactions, leading to slow, labor-intensive processes prone to errors. These challenges highlight the need for more efficient, automated approaches to enhance diagnostic accuracy.

Deep learning, particularly convolutional neural networks (CNNs), has revolutionized the field of medical image analysis. CNNs are proficient at learning intricate patterns from data, making them highly effective in segmenting and classifying nuclei, even under difficult conditions. These networks excel in extracting relevant features such as nucleus shape, size, and texture, enabling accurate tissue analysis. However, while CNNs have shown great promise, existing techniques still face limitations in handling complex cases, such as overlapping nuclei and varying tissue morphologies, especially in diseases like DLBCL.

HoVerNet, an advanced deep learning architecture, offers a promising solution to these challenges. This method employs multi-branch processing to perform both segmentation and classification simultaneously, making it highly effective for the analysis of clustered, heterogeneous nuclei. HoVerNet’s ability to extract precise morphological features, such as nuclear area, perimeter, and shape, allows for more accurate tumor cell quantification. It also opens the door to the development of new prognostic markers and improvements in diagnostic accuracy, addressing the gaps left by traditional methods.

This research aims to leverage HoVerNet’s capabilities to enhance the diagnosis, subtyping, and severity assessment of DLBCL. By addressing the current limitations in medical imaging techniques, this approach promises to improve the precision and efficiency of DLBCL analysis, aiding pathologists in making better-informed clinical decisions. The innovation lies in combining deep learning with tissue image analysis to create a more automated, accurate, and clinically useful framework for cancer diagnosis.

## 2. Literature Review

Image patches are small, square regions extracted from larger medical images for tasks such as region of interest (ROI) identification, feature extraction, and AI algorithm applications. They enhance analysis by focusing on specific areas, such as structures and textures, with patch sizes varying from single pixels to predefined windows. Basu et al. [2] captured 500 DLBCL and non-DLBCL tissue images at 40× magnification using microscope-based cameras, while El Hussien et al. [3] analyzed 256 × 256 patches from digitally stained H&E slides of CLL, aCLL, and RT cases. Wójcik et al. [4] standardized 37,665 H&E images of DLBCL lymph nodes to 448 × 448 pixels, and Li et al. [5] captured 400× magnification images from 500 labeled DLBCL tissue sections. Swiderska-Chadaj et al. [6] digitized 42 H&E DLBCL slides for external validation, and Bándi et al. [7] extracted annotated patches from six tissue types using WSIs. Shankar et al. [8] analyzed classic Hodgkin lymphoma, mantle cell lymphoma, and DLBCL cores at 40× magnification, while Swiderska-Chadaj et al. [9] derived 512 × 512 patches at 5× magnification for training. Perry et al. [10] applied a self-supervised phase on FFPE H&E-stained biopsy WSIs of aggressive B-cell lymphoma, dividing 20× or 40× images into 384 × 384 patches for analysis.

Pre-processing prepares image data for model input, reducing training time and improving inference. Techniques include orientation, resizing, grayscale conversion, and exposure adjustments to enhance image quality and feature extraction. Hamdi et al. [11] applied Gaussian filters, Laplacian filters, color normalization, and Gradient Vector Flow for feature extraction. Vrabac et al. [12] used tissue microarrays for cell nucleus extraction from H&E-stained images, while Basu et al. [2] developed attention map transformers and feature fusion for DLBCL classification. Blanc-Durand et al. [13] employed resampling, padding, cropping, and adaptive thresholding on PET and CT data to extract tumor heterogeneity features. Ferrández et al. [14] used Gaussian filtering, metabolic tumor volume, and standard uptake value metrics to analyze tumor dissemination. El Hussien et al. [3] annotated ROIs and measured nuclear contour and hull areas, while Graham et al. [15] applied Otsu thresholding, color adjustments, and textural feature extraction. Ferrández et al. [16] and Mohlman et al. [17] utilized normalization, filtering, max-pooling, ReLU operations, and edge detection in pre-processing workflows. Other studies [8,9,10,18,19,20,21] applied normalization, machine learning algorithms, filtering, and feature selection for enhanced image quality and accurate DLBCL classification.

Deep learning, especially CNNs, is highly effective in processing and classifying medical images, such as distinguishing healthy and cancerous cells in DLBCL. HoVerNet excels in nuclei segmentation and classification by integrating segmentation and classification branches, leveraging nuclear pixel distances, and enabling complex pathology analysis. Vrabac et al. [12], El Hussein et al. [3], Wójcik et al. [4], and Graham et al. [15] applied or trained HoVerNet for various tasks, with training epochs ranging from 50 to 800. Hamdi et al. [11] achieved superior performance using MobileNet-VGG-16 with an AUC of 99.43% and 99.8% accuracy on 15,000 H&E-stained WSIs. Blanc-Durand et al. [13] and Ferrández et al. [14,16] utilized 3D U-Net with Adam optimization for PET/CT scans, while Swiderska-Chadaj et al. [6] and Bándi et al. [7] also employed U-Net. Additionally, Basu et al. [2] and Vrabac et al. [12] used DenseNet-201 and ResNet-50, respectively, with optimized training parameters for DLBCL analysis. A study evaluating 17 CNN models (including VGG16) across three hospitals reported average patch-level diagnostic accuracy between 87–96%, demonstrating strong performance in DLBCL vs. non-DLBCL differentiation [5].

However, there are still challenges in nuclei segmentation and classification for DLBCL that need to be addressed. Many studies do not work well with different staining types like CMYC and H&E, which limits their use. Most research also does not focus on adding these tools into real clinical workflows or electronic medical records (EMRs), which are important for practical use. Scalability and real-time analysis are also not well explored, as many methods are not designed for large-scale use. This study helps solve these problems by showing that HoVerNet performs better on CMYC-stained images, creating a simple GUI.

## 3. Materials and Methods

**Figure 1:**
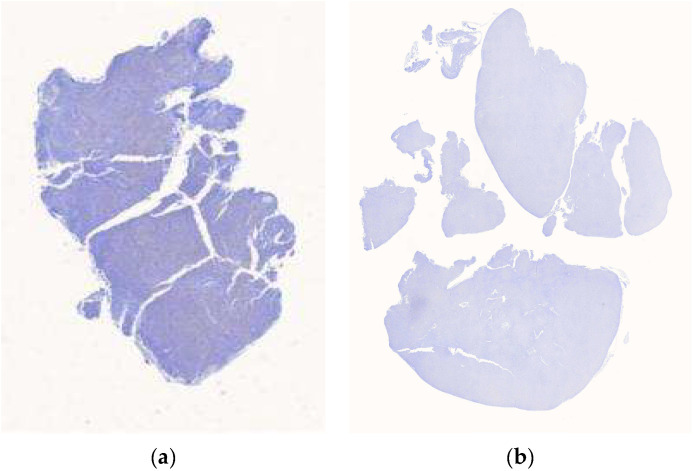
Examples of CMYC-stained whole slide images. (**a**) MYC+ whole slide image; (**b**) MYC˗ whole slide image.

A total of 122 digital WSIs of DLBCL, comprising 61 MYC+ cells and 61 MYC- cells, were collected from the Department of Pathology, Hospital Universiti Sains Malaysia (Hospital USM). These WSIs were scanned at 40× magnification using the Motic EasyScan Pro digital slide scanner. The collection and use of these images followed ethical guidelines outlined in the Declaration of Helsinki. Approval was obtained from the Jawatankuasa Etika Penyelidikan Manusia Universiti Sains Malaysia (JEPeM-USM), under the reference USM/JEPeM/22110749, ensuring compliance with ethical and legal standards. Only CMYC-stained images were used in this study due to the availability and standardization of these samples within the dataset. While this ensures consistency in analysis and training, it limits the model’s generalizability to other staining protocols, such as H&E. Figure 1 illustrates the examples of CMYC-stained whole slide images.

### 3.1. Lossless Image Compression

Lossless image compression is a type of image compression method that reduces the image file size without losing any important information [22]. The process begins by loading the image using the ‘PIL’ library [23]. The image is opened and identified using the ‘Image.open()’ function. Once the image is loaded, the next step is to resize it by using the ‘resize()’ function, which takes two parameters: the new size of the image and the resampling filter. The new size is taken by modifying the value of the resize factor, by which both width and height are divided by the resize factor. The resampling filter used is the ‘Image.LANCZOS’ function, which is known for producing high-quality results. The function is nonzero only within the interval (−1 < *x* < 1). After resizing, the image is saved to the specified output path using the ‘save()’ function. Lossless image compression reduces the file size of images without sacrificing important information, preserving data quality for analysis. This is achieved using the LANCZOS resampling filter, which provides high-quality results by smoothing the image while retaining sharpness and detail. This method ensures that the compressed images maintain essential features needed for segmentation and classification tasks. By resizing images, computational requirements for model training and inference are significantly reduced, enabling faster processing while retaining the integrity of nuclei features. The Lanczos function is defined as in (1).

(1) $$(x)=\left\{\begin{array}{c} \frac{1}{2}((2-\left|x\right|)sin(\pi x)+sin(2\pi x)),\;\;\;if\;\left|x\right|<1\;\\ 0\;\;\;\;\;\;\;\;\;\;\;\;\;\;\;\;\;\;\;\;\;\;\;\;\;\;\;\;\;\;\;\;\;\;\;\;\;\;\;\;\;\;\;\;\;\;\;\;\;\;\;\;\;\;,\;otherwise \end{array}\right.$$

### 3.2. Image Patches

**Figure 2:**
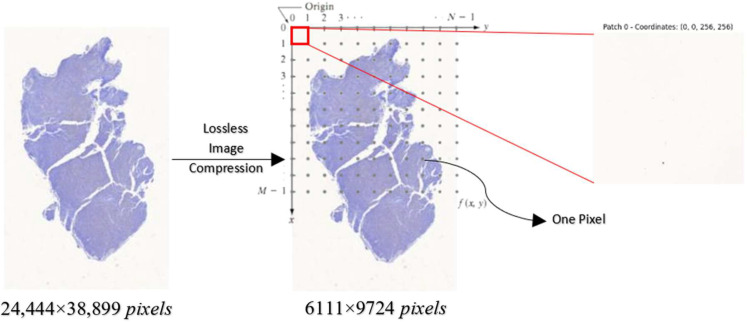
Flow process of image patches.

Image patching is a crucial technique for localizing ROI, extracting features, reducing computational complexity, and enhancing accuracy in analyzing DLBCL images [24]. By breaking down these images into smaller patches, specific ROIs like DLBCL nuclei can be localized and analyzed. These patches allow the computation of various attributes such as color, texture, gradients, and shapes. Analyzing patches rather than the entire image reduces computational complexity, making segmentation and classification algorithms more manageable. Additionally, focusing on smaller regions allows algorithms to better handle variations in intensities, shapes, or textures, potentially leading to more accurate segmentation results. The formula for image patching is expressed as in (2). Figure 2 depicts the flow process of image patches. The original WSI, with dimensions of 24,444 × 38,899 pixels, undergoes lossless image compression, reducing its dimensions to 6111 × 9724 pixels. Following compression, the resized image is divided into square patches during the image patching process, with each patch measuring 256 × 256 pixels.

(2) K=(M−W+1)×(N−H+1)   

### 3.3. Normalization

Normalization is a crucial preprocessing step in medical image analysis, particularly for nuclei segmentation and classification. It enhances image quality, reduces distortions, and adjusts pixel intensity values to a consistent range [25], typically between 0 (black) and 1 (white). Normalization involves identifying and outlining the cell containing ROI within the image. This ensures better feature extraction by mitigating noise from lighting or staining variations. For grayscale images, normalization is applied to a single channel, while for RGB images, it is applied to all three channels. This step improves model stability, generalization, and accuracy, especially when dealing with dark, unevenly illuminated patches or datasets with diverse staining techniques. The normalization process is described as in (3).

(3) $$z_{i}=\frac{x_{i}-min(x)}{max(x)-min(x)}\;$$

### 3.4. CNN Architecture

**Figure 3:**
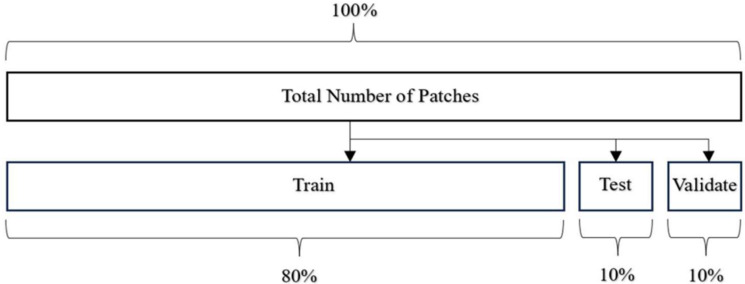
Training, validation, and testing scheme.

The dataset was split into 80% training, 10% testing, and 10% validation subsets after patch extraction, not by whole slide images. This ensured a diverse and balanced distribution of input images for the CNN models. This distribution ensures sufficient data for model optimization while retaining samples for unbiased evaluation and fine-tuning (Figure 3). HoVerNet was chosen because it is specifically designed for both nuclei segmentation and classification, which is important for DLBCL analysis [4,11,15]. Its encoder-decoder structure with residual units improves feature extraction and segmentation accuracy [11]. The up-sampling branches further enhance classification by precisely predicting nucleus types, enabling the network to handle clustered and morphologically diverse nuclei in DLBCL WSIs. This study enhances the original HoVerNet framework by adapting it for CMYC-stained WSIs of DLBCL, addressing morphological and staining variations not considered in the original model. The architecture is modified for binary classification of MYC+ and MYC˗ nuclei, aligned with clinical relevance in lymphoma assessment. Model performance is optimized through systematic hyperparameter tuning, and a diagnostic scoring method based on MYC expression is introduced to estimate disease severity. Based on the literature, it performs better than U-Net and ResNet. U-Net is effective for segmentation but does not include classification, making it less suitable for identifying nucleus types [15]. ResNet is good for general image classification but lacks features needed for detailed segmentation of overlapping nuclei, which is common in DLBCL tissue images. A multi-class deep learning study using VGG16 successfully distinguished between benign nodes, DLBCL, Burkitt lymphoma, and small lymphocytic lymphoma with 95% accuracy [26]. These findings from previous studies guided the decision to use HoVerNet and to compare with VGG16 in this work. A GUI is developed to enable real-time segmentation, cell counting, and severity visualization, supporting practical integration into clinical workflows.

**Figure 4:**
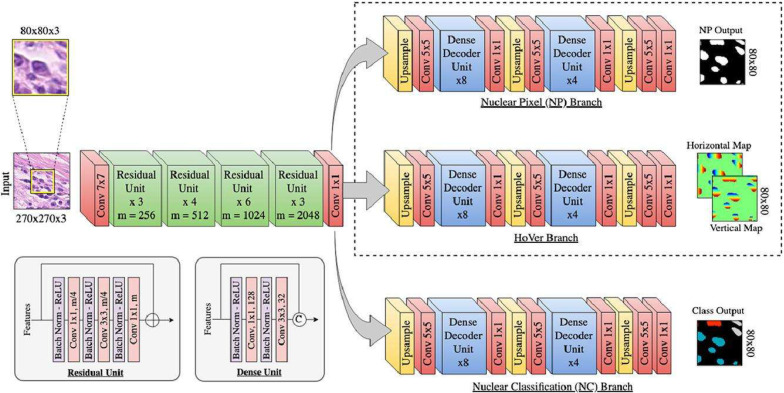
HoVerNet architecture [11].

Figure 4 illustrates the HoVerNet architecture [11]. The model was trained for 800 epochs to ensure comprehensive learning of complex patterns in DLBCL datasets [4]. The model was implemented using PyTorch version 2.2.0, with minor modifications to support binary classification of MYC+ and MYC- nuclei. The training used the Adam optimizer with a learning rate of 1 × 10^−4^ and a batch size of 32 and 64. The binary cross-entropy loss function was applied to optimize performance for binary classification. Patch size was 256 × 256 pixels as described in Section 3.2. This choice was guided by the high complexity of the data, requiring extended training to capture intricate features [4]. Early stopping, a common technique to prevent overfitting, was considered but not implemented in this study. Instead, overfitting was managed by closely monitoring validation loss and using batch size adjustments.

### 3.5. Nuclei Counting and Diagnosis of Severity Level of Cells

After segmenting and classifying nuclei using HoVerNet in DLBCL images, nuclei counting is proposed. The segmented image identifies and labels each nucleus based on its type: normal cells are smaller (average 36.7 pixels), appearing dark blue; positive cancer cells are larger (average 73.5 pixels), lighter blue; negative cancer cells are also large (average 73 pixels), with a lighter blue hue. Cell counting involves using connected component labeling algorithms to distinguish and count these nucleus types. The counts are validated against manual or other automated methods. Subsequently, totals of abnormal (negative and positive) and all cells (normal, negative, and positive) are calculated. Percentages of negative and positive cells relative to total cells are then computed using a formula as in (4).

(4) $$Percentage=\frac{Negative\;Count+Positive\;Count}{Total\;Count}\times 100\%$$

Disease severity is assessed based on nucleus characteristics like size, shape, color, and abnormalities, with each cell assigned a severity score. Cells exceeding 40% in abnormality are classified as ‘Severe’; otherwise, they are ‘Mild’. This process aids in diagnosing and monitoring disease progression in DLBCL. The 40% threshold was determined through consultation with clinical pathologists and reflects a typical cutoff observed in high-grade cases of DLBCL where MYC-positivity exceeds this level [27].

### 3.6. Graphic User Interface (GUI)

The data collection and preparation begin with a GUI where patient details like Patient ID and Year of Data are input to associate image analysis with the correct patient records. Once validated, the user selects an image file (PNG, JPG, JPEG, BMP, GIF) for analysis. The selected image and its patches are normalized to enhance contrast and displayed for inspection. Nuclei detection follows, converting images to grayscale if needed and applying binary thresholding for nuclei segmentation (‘Nuclei Pixel Branch’) visualized on the GUI.

Gradient computation (‘HoVer Branch’) highlights textural patterns and directional changes, aiding detailed feature analysis. Nuclei are segmented and classified based on predefined criteria, visualized to show categorization into ‘Negative Cells’, ‘Positive Cells’, and ‘Normal Cells’. Cell counting follows, tallying each type and computing total counts. The proportion of negative and positive cells is used to determine disease severity (‘Severe’ or ‘Mild’). Results, including counts, percentages, and severity assessments, are displayed on the GUI for comprehensive analysis. A ‘Finish’ button concludes analysis, confirming the completion of cell counting.

## 4. Results and Discussion

### 4.1. Image Pre-Processing

DLBCL WSIs while maintaining image quality. Using the LANCZOS filter, the resizing process preserved a resolution of 96 dpi and a bit depth of 24 bits, ensuring clarity and color fidelity. This reduction effectively minimizes computational overhead, facilitating more efficient downstream processing. Following compression, image patching yielded 34,000 distinct 256 × 256-pixel patches. Each patch was uniquely named based on its coordinates within the original image and systematically stored. Visualization of these patches revealed diverse pathological features, improving granularity for deep learning applications. This structured patch extraction enhances the deep learning model’s ability to focus on specific tissue regions within DLBCL images, optimizing feature learning for classification and analysis. Normalization of the extracted patches further improved consistency by scaling pixel intensity values to the [0,1] range, achieved through division by 255. This transformation mitigated variability in raw pixel values, leading to improved model stability and faster convergence during training. Standardization plays a crucial role in optimizing deep learning performance, ensuring more accurate and efficient analysis of DLBCL images.

### 4.2. Performance Evaluation of HoVerNet Optimization Results

Before feeding the images into HoVerNet and VGG16, the dataset is divided into training, testing, and validation sets (Table 1).

**Figure 5:**
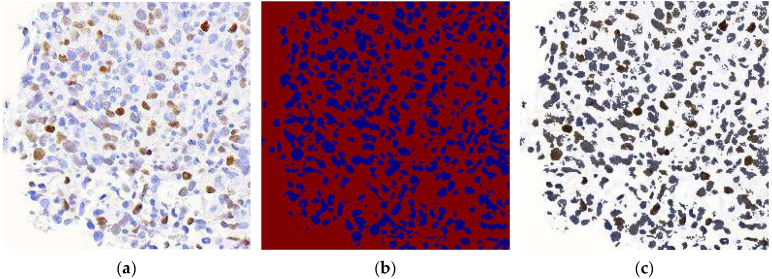
Nuclei Pixel Branch of HoVerNet. (**a**) Normalized Patched Image; (**b**) Nuclei Pixel Branch; (**c**) Overlaid Nuclei Image.

HoVerNet consists of three branches: the ‘Nuclei Pixel Branch,’ the ‘HoVer Branch,’ and the ‘Nuclei Classification Branch.’ The process starts with normalized patched images as input. These images are processed to detect nuclei, producing a binary image where nuclei pixels are set to one value (blue), and non-nuclei pixels are set to another value (red), as shown in Figure 5b. The output from the ‘Nuclei Pixel Branch’ is then overlaid on the normalized patched image, creating a composite image. In this overlay, the nuclei are distinctly highlighted against the background, making them easier to visually identify and analyze.

**Figure 6:**
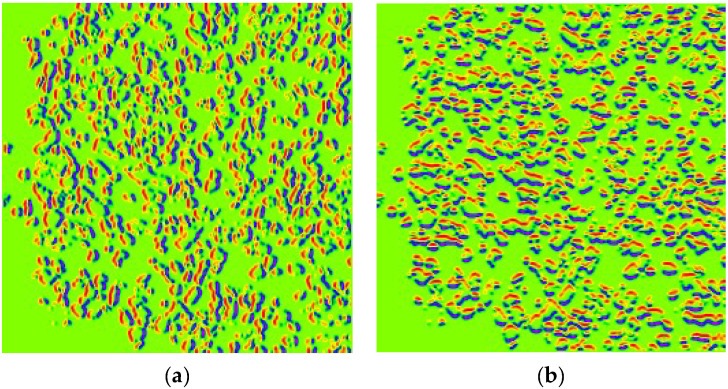
HoVer Branch of HoVerNet. (**a**) Horizontal Map Channel; (**b**) Vertical Map Channel.

The overlay image is then projected onto the horizontal and vertical axes to create horizontal and vertical images, respectively. This process introduces an additional branch called the ‘HoVer Branch’ (Figure 6). These projections provide useful summaries of the spatial distribution of the nuclei in the image. For example, a horizontal projection provides information on how the nuclei are distributed from top to bottom, while a vertical projection provides information on how they are distributed from left to right.

After defining the architecture, an instance of the HoVerNet model was created and compiled using the Adam optimizer and binary cross-entropy loss function, suitable for binary classification tasks. The model was initially trained for 800 epochs with batch sizes of 32 and 64, following the approach of Wójcik et al. [4], who reported achieving the highest F1 score of 0.939 for similar tasks.

**Figure 7:**
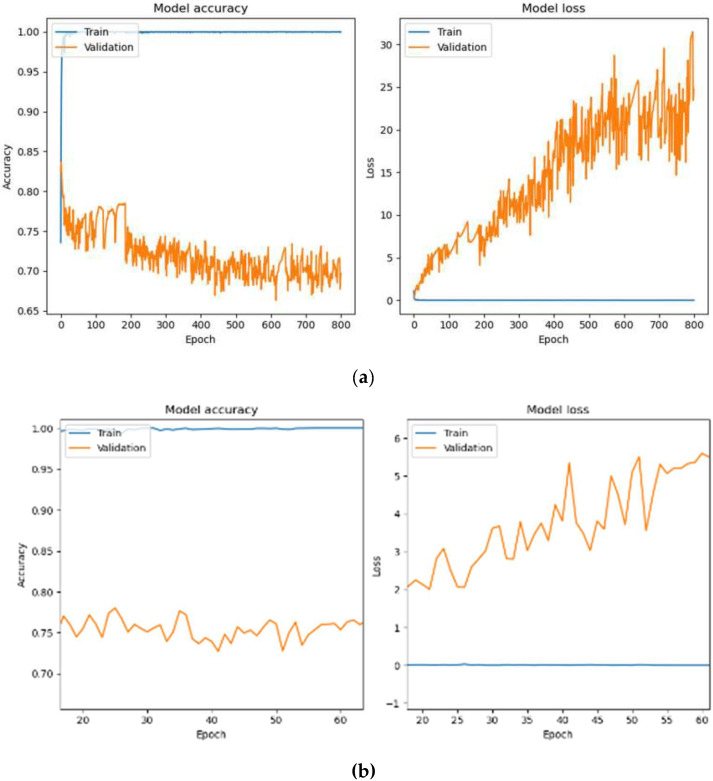
Accuracy-loss graph for 800 epochs with Adam optimizer (Batch size 32). (**a**) Full accuracy-loss graph for epochs 0–800; (**b**) Zoomed analysis for epochs 20–60.

Figure 7a depicts the training process of the HoVerNet model over 800 epochs with a batch size of 32. The model achieved perfect training accuracy of 100% and an exceptionally low training loss of 2.21 × 10^−4^. However, validation and testing accuracy were notably lower, at 69.63% and 71.26%, with higher validation and testing losses at 24.7998 and 23.0890, respectively. These results indicate overfitting, as the model performed well on training data but failed to generalize effectively to unseen data. Figure 7b focuses on the training dynamics for a batch size of 32 over a zoomed-in epoch range of 20–60. While the training accuracy was consistently 100%, validation performance remained unstable, showing fluctuations in accuracy and higher validation losses. This pattern indicates overfitting, as the model was overly tuned to the training data and struggled to generalize to unseen data.

**Figure 8:**
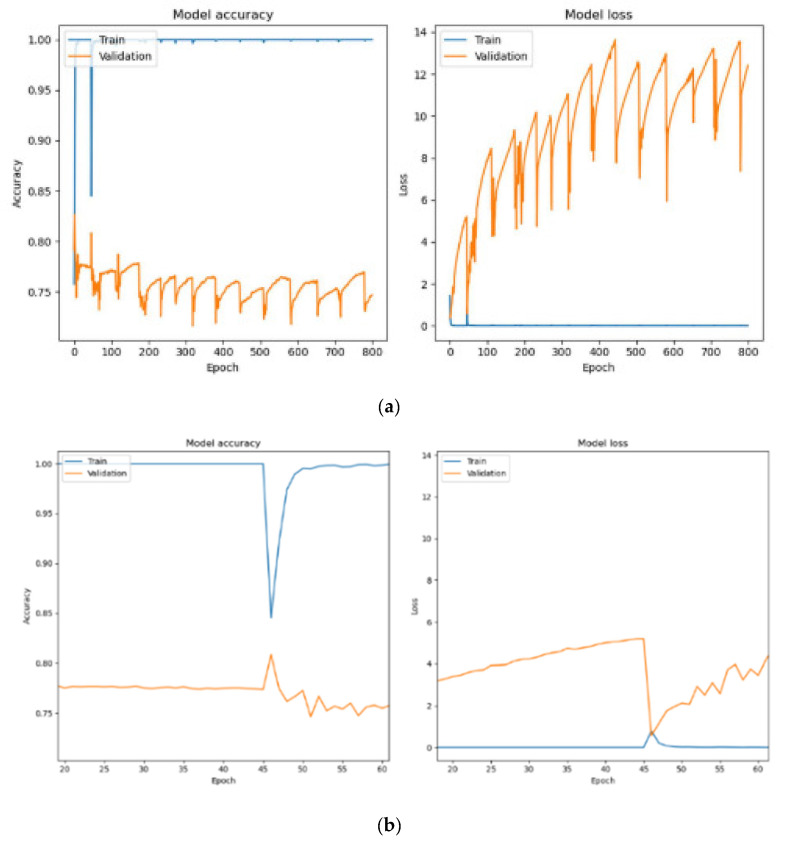
Accuracy-loss graph for 800 epochs with Adam optimizer (Batch size 64). (**a**) Full accuracy-loss graph for epochs 0–800; (**b**) Zoomed analysis for epochs 20–60.

Figure 8a shows the training and validation performance when using a batch size of 64. The model achieved 100% training accuracy, with an even lower training loss of 1.07 × 10^−7^. Validation and testing accuracies improved to 74.68% and 75.75%, respectively, with reduced validation losses of 12.4109 and testing loss of 11.9979. The larger batch size resulted in more stable training, with fewer parameter updates per epoch, enabling better generalization to unseen data.

Figure 8b provides a closer analysis of training with a batch size of 64 between epochs 20 and 60. A sharp decline in training accuracy occurred at epoch 45 due to adjustments in model parameters to balance training and validation losses. After this drop, the training accuracy quickly recovered and stabilized at 100%. Validation accuracy showed steady improvement, and validation losses decreased over time, demonstrating the model’s ability to generalize effectively. However, after epoch 45, the accuracy of validation gradually decreases as the epoch increases. This indicates that HoVerNet achieves better performance and generalization when trained for fewer epochs (less than 45).

Further investigation into the optimal number of epochs (15, 20, 25, 30, 35, 45, and 50) aimed to maximize validation and testing accuracies while minimizing losses (Table 2). For batch size 32, the model reached 100% training accuracy by epoch 35, with validation and testing accuracy peaking at 82.78% and 83.67%, respectively. Beyond this, accuracy declined, indicating overfitting. At epoch 100, training accuracy decreased to 98.72%, with further drops in validation and testing accuracies, suggesting underfitting and potential learning rate issues.

**Figure 9:**
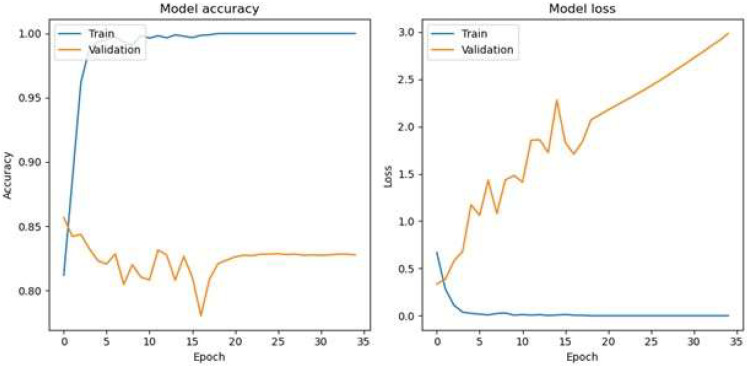
Accuracy loss graph of epoch 35 with Adam optimizer (Batch size 32).

**Figure 10:**
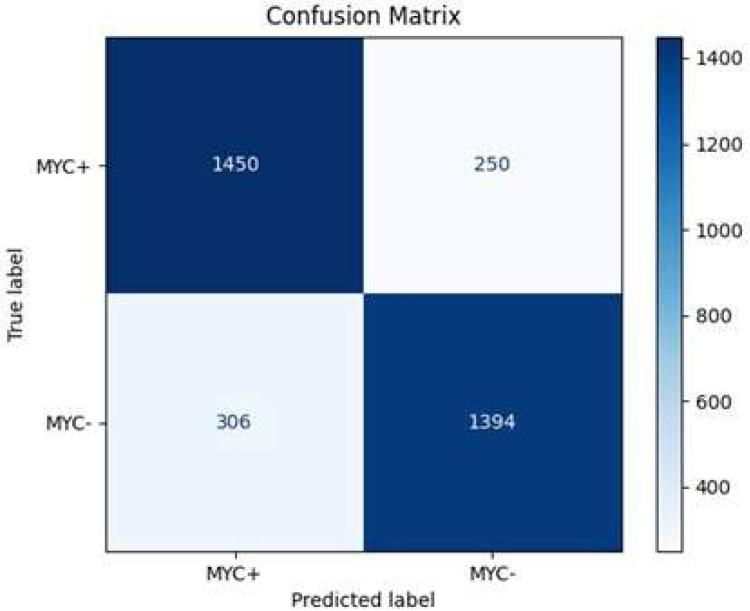
Confusion matrix of epoch 35 with Adam optimizer (Batch size 32).

The optimal epoch for a batch size of 32 for the HoVerNet and VGG16 models is 35 (Figure 9), where validation and testing accuracies were highest, balancing effective generalization and minimizing overfitting. This iterative approach allowed for fine-tuning the model’s training dynamics and enhancing its ability to generalize effectively. Based on the confusion matrix in Figure 10, the HoVerNet model correctly identified 1450 MYC+ instances and 1394 MYC- instances, resulting in a high true positive (TP) and true negative (TN) count. However, it misclassified 250 MYC- instances as MYC+ (FP) and 306 MYC+ instances as MYC- (FN).

Based on Table 2 and Table 4, VGG16 achieved higher classification accuracy compared to HoVerNet. However, VGG16 is a standard CNN not inherently designed for nuclei segmentation tasks. It performs well in distinguishing image-level classes but lacks the ability to accurately delineate individual nuclei, especially in cases of overlapping structures. In contrast, HoVerNet is specifically optimized for nuclear instance segmentation and classification, making it more adept at handling the complex morphological variations and overlapping nuclei commonly seen in histopathological images of DLBCL. Although its overall accuracy is lower than VGG16, HoVerNet offers superior segmentation granularity and biological interpretability, which are essential for meaningful diagnostic assessment in clinical practice.

These results are tabulated in Table 3, with an overall accuracy of approximately 85.2%, indicating that the model is effective in its predictions. The precision, which measures the accuracy of positive predictions, is about 85.3%, while the recall, reflecting the model’s ability to identify true positive cases, stands at 82.6%. The specificity, or true negative rate, is 84.8%, highlighting the model’s proficiency in correctly identifying negative instances. Additionally, the F1 score, a balance between precision and recall, is around 83.9%, underscoring the model’s robustness in handling both MYC+ and MYC- classification.

**Figure 11:**
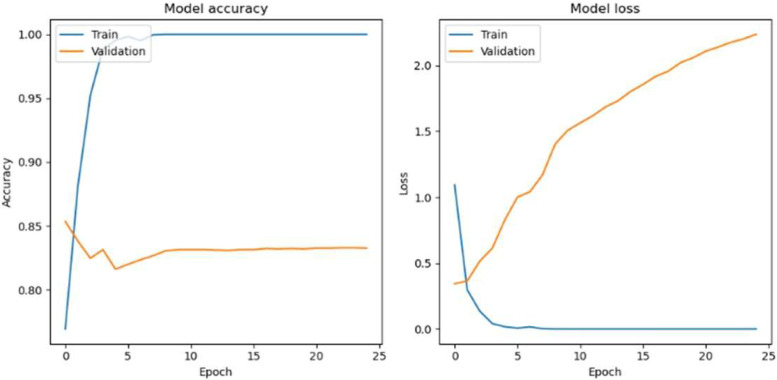
Accuracy loss graph of epoch 25 with the Adam optimizer (Batch size 64).

Apart from that, when the Adam optimizer with a batch size of 64 is used, the HoVerNet model achieves a training accuracy of 100% across all epochs (Table 4), indicating that it has perfectly learned the training dataset. However, this does not translate to the validation and testing sets, where the accuracy is significantly lower, suggesting that the model is overfitting to the training data. In the early epochs, there is a gradual increase in both validation and testing accuracies, peaking at 83.25% and 84.46%, respectively, at epoch 25 (Figure 11). This could be the model’s sweet spot, where it has learned enough patterns to generalize well to unseen data. Beyond this point, there is a noticeable decline in performance on the validation and testing sets, with the lowest accuracy observed at epoch 100, dropping to 74.79% and 74.53%, respectively. This decline could be due to the model becoming too specialized in the training data features, which do not represent the broader patterns needed for new data.

**Figure 12:**
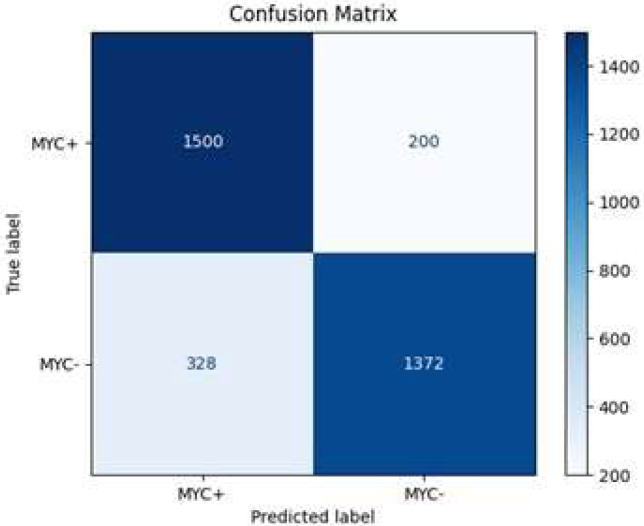
Confusion matrix of epoch 25 with the Adam optimizer (Batch size 64).

The model correctly identified 1500 MYC+ instances and 1372 MYC- instances, resulting in a high true positive (TP) and true negative (TN) count. However, it misclassified 200 MYC- instances as MYC+ (FP) and 328 MYC+ instances as MYC- (FN) (Figure 12). These metrics indicate that the model achieves an overall accuracy of approximately 85.5% (Table 5), demonstrating its reliability in predicting MYC+ and MYC- cases. The precision of 82.1% reflects the model’s accuracy in predicting positive cases, while the recall of 88.2% indicates a high true positive rate. The specificity, at 80.7%, shows the model’s effectiveness in correctly identifying negative cases. The F1 score of 85.1% balances both precision and recall, emphasizing the model’s robustness in classification tasks.

Comparing batch sizes of 32 and 64 shows significant differences in performance metrics and execution times. Although both batch sizes achieve perfect training accuracy, a batch size of 64 yields higher validation and testing accuracy with lower losses, and it reaches these results in fewer epochs (25 vs. 35). Additionally, training time per epoch is slightly shorter for a batch size of 64 (62 s vs. 63 s), and validation and testing times are also faster, taking 7 s compared to 8 s for batch size 32. These findings suggest that a batch size of 64 provides better efficiency and faster convergence.

**Figure 13:**
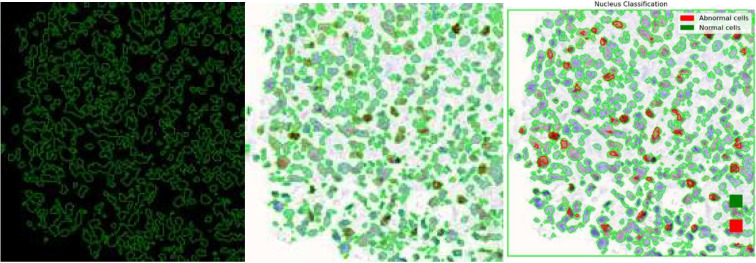
Simultaneous nuclei segmentation and classification (Nuclei Classification Branch of HoVerNet).

After model training, the simultaneous nuclei segmentation and classification process is taken. This process is defined as the last branch in HoVerNet, known as the ‘Nuclei Classification Branch.’ HoVerNet contains an encoder-decoder structure that can capture both high-level and low-level features in the images, which helps in accurately segmenting an image. Abnormal cells (positive cells and negative cells) are classified in red while normal cells are classified in green (Figure 13).

### 4.3. Nuclei Counting and Diagnosis of Severity Level of the Cells

In the diagnosis of cellular abnormalities, the severity of cell changes can be categorized into two levels: mild and severe. Based on the nuclei count results generated via automated analysis, it was found that the dataset primarily consists of normal cells, positive cells, and negative cells. Specifically, in one of the examples for MYC+, ‘U58-20-2 B042920 CMYC’, there were 8371 negative cells and 16,381 positive cells identified, contributing to a total of 24,752 abnormal cells. In contrast, 9971 cells were classified as normal. The analysis encompassed a total of 34,723 cells. The percentage of abnormal cells, consisting of both negative and positive types, amounted to 71.28% of the total cell population. This high percentage categorizes the dataset as severe, indicating a substantial presence of abnormal cellular characteristics. Table 6 tabulates the results of cell counting and the severity level of each cell in MYC+. Besides, Table 7 tabulates the results of cell counting and the severity level of each cell in MYC-.

### 4.4. Graphic User Interface (GUI)

The Tkinter-based application for DLBCL nuclei segmentation and classification allows users to input patient information, upload an image, visualize image patches, and analyze cells. Initially, a main window was created for inputting patient data. If the patient’s ID and year are not filled in, an error message appears. Once an image is selected, it is displayed in the window with an option to view patch images. Users can normalize the image, convert it to grayscale, and create a binary image highlighting the nuclei. Gradient images, showing horizontal and vertical gradients, can also be displayed.

**Figure 14:**
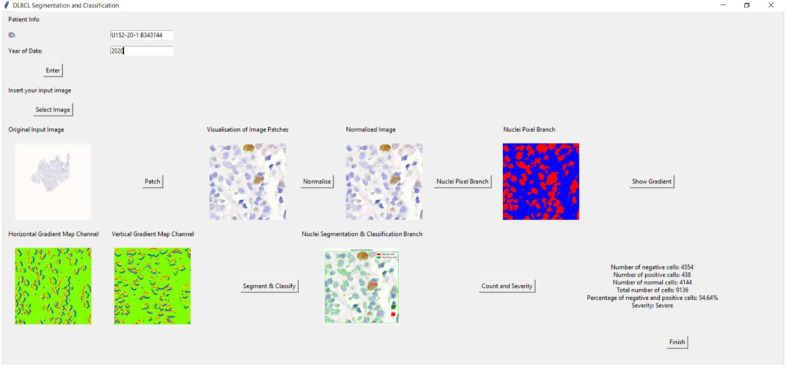
Overview of GUI for DLBCL diagnosis.

The application further segments and classifies nuclei, enabling cell counting. It calculates the number of negative, positive, and normal cells, their percentages, and the severity based on these percentages. Results are displayed, and a ‘Finish’ button concludes the process, providing a diagnostic tool for medical professionals. This application streamlines the process of analyzing DLBCL nuclei, making it efficient and user-friendly for medical use. Figure 14 shows the overview of the GUI for DLBCL diagnosis. As an example of the GUI in action, one clinical slide was analyzed through the full pipeline. The system detected 4554 negative cells, 438 positive cells, 4114 normal cells, and a total of 9136 cells. It identified 54.64% as abnormal and classified the case as ‘Severe,’ demonstrating the practical functionality and diagnostic potential of the application.

## 5. Conclusions

This study analyzed 122 digital high-magnification WSIs of DLBCL using advanced pre-processing and the HoVerNet deep learning model. The model successfully classified nuclei, automated cell counting, and assessed disease severity. These improvements make pathology workflows faster and more accurate. A GUI was also developed, making it easier for pathologists to use the system.

However, there are some limitations. HoVerNet’s complex multi-branch architecture led to overfitting, as observed by a sharp performance decline after epoch 45. This overfitting suggests that the model was overly complex for the dataset, performing well on training data but poorly on unseen data. Future work should include optimizing hyperparameters such as dropout, data augmentation, integrating attention mechanisms to improve feature selection and reduce overfitting, and exploring alternative architectures. By addressing these issues, the system can improve DLBCL diagnosis and make pathology workflows faster, more accurate, and easier to use in different medical settings.

## Figures and Tables

**Table 1:** Dataset Distribution.

Class	Dataset Distribution		
	Training (80%)	Testing (10%)	Validation (10%)
MYC+	13,600	1700	1700
MYC-	13,600	1700	1700
Total	27,200	3600	3600

**Table 2:** Accuracies and losses for each epoch with Adam optimizer (Batch size 32).

Epochs	HoVerNet					VGG16						
	Acc	Loss	Acc	Loss	Acc	Loss	Acc	Loss	Acc	Loss	Acc	Loss
	Training		Validation		Testing		Training		Validation		Testing	
15	99.79	2.25 × 10^−2^	80.37	1.8699	81.22	1.747	98.89	2.86 × 10^−2^	92.29	0.2787	93.62	0.2328
20	99.99	4.47 × 10^−4^	80.01	2.6568	80.37	2.5758	92.30	1.23 × 10^−2^	97.48	0.379	93.11	0.1654
25	99.97	1.90 × 10^−3^	75.00	2.7742	75.94	2.5074	93.80	9.8 × 10^−3^	94.73	0.0354	95.32	0.2298
30	99.85	7.60 × 10^−3^	78.48	1.8445	78.21	1.782	100.00	2.09 × 10^−1^	94.92	0.105	98.67	0.1346
**35**	**100.00**	**7.86 × 10^−9^**	**82.78**	**2.9870**	**83.67**	**2.7953**	**100.00**	**2.42 × 10^−2^**	**100.00**	**0.0272**	**99.87**	**0.0529**
45	100.00	6.03 × 10^−9^	80.66	5.6762	81.01	5.4998	97.65	4.97 × 10^−2^	98.54	0.0401	98.87	0.3312
50	100.00	1.27 × 10^−7^	78.86	4.1112	80.75	3.5631	97.47	7.56 × 10^−2^	91.56	0.4981	92.44	0.4114
100	98.72	6.16 × 10^−2^	76.47	3.4293	78.45	3.1200	-	-	-	-	-	-
800	100.00	2.21 × 10^−4^	69.63	24.7998	71.26	23.089	-	-	-	-	-	-

Bold: Best accuracy and loss results for HoVerNet and VGG16.

**Table 3:** Testing model performance results with Adam optimizer (Batch size 32).

Epochs	Accuracy	Loss	MYC+	MYC˗	MYC+	MYC˗	MYC+	MYC˗
			F1 Score		Recall		Precision	
15	81.22%	1.7470	0.81	0.81	0.82	0.80	0.81	0.82
20	80.37%	2.5758	0.81	0.80	0.82	0.78	0.79	0.82
25	75.94%	2.5074	0.76	0.76	0.76	0.75	0.76	0.76
30	78.21%	1.7820	0.79	0.78	0.79	0.78	0.78	0.79
**35**	**83.67%**	**2.7953**	**0.84**	**0.83**	**0.85**	**0.82**	**0.83**	**0.85**
45	81.01%	5.4998	0.81	0.81	0.82	0.80	0.80	0.82
50	80.75%	3.5631	0.81	0.80	0.82	0.79	0.80	0.82
100	78.45%	3.1200	0.79	0.78	0.79	0.77	0.78	0.79
800	71.26%	23.0890	0.72	0.71	0.74	0.69	0.70	0.72

Bold: Best testing model results with Adam optimizer (Batch size 32).

**Table 5:** Testing model performance results with the Adam optimizer (Batch size 64).

Epochs	Accuracy	Loss	MYC+	MYC˗	MYC+	MYC˗	MYC+	MYC˗
			F1 Score		Recall		Precision	
15	83.43%	1.7470	0.83	0.84	0.82	0.85	0.84	0.83
20	83.87%	2.5758	0.84	0.84	0.82	0.86	0.85	0.83
**25**	**84.46%**	**2.5074**	**0.85**	**0.84**	**0.85**	**0.84**	**0.84**	**0.85**
30	83.34%	1.7820	0.83	0.84	0.82	0.84	0.84	0.83
35	79.92%	2.7953	0.79	0.78	0.79	0.77	0.78	0.79
45	81.87%	5.4998	0.82	0.82	0.82	0.81	0.82	0.82
50	82.52%	3.5631	0.82	0.83	0.82	0.83	0.83	0.82
100	74.53%	3.1200	0.75	0.74	0.76	0.73	0.74	0.76
800	75.75%	11.9979	0.72	0.71	0.74	0.69	0.70	0.72

Bold: Best testing model performance results with Adam optimizer (Batch size 64).

**Table 6:** Results of nuclei counting and severity level (MYC+).

MYC+ Slide	Negative Cells	Positive Cells	Abnormal Cells (Negative + Positive)	Normal Cells	Total Number of Cells	Percentage of Abnormal Cells	Severity Level
Slide 1	22,992	45,887	68,879	10,537	79,416	86.73%	Severe
Slide 2	32,246	64,706	96,952	41,867	138,819	69.84%	Severe
Slide 3	9941	13,819	23,760	2464	26,224	90.60%	Severe
Slide 4	20,481	59,398	79,879	29,734	109,613	72.87%	Severe
Slide 5	8272	43,783	52,055	11,561	63,616	81.83%	Severe
Slide 6	7815	25,173	32,988	8754	41,742	79.03%	Severe
Slide 7	9646	21,653	31,299	12,799	44,098	70.98%	Severe
Slide 8	10,556	83,276	93,832	49,288	143,120	65.56%	Severe
Slide 9	5882	37,853	43,735	17,402	61,137	71.54%	Severe
Slide 10	22,351	27,618	49,969	8896	58,865	84.89%	Severe
Slide 11	45,300	85,022	130,322	40,412	170,734	76.33%	Severe
Slide 12	30,211	60,296	90,507	17,320	107,827	83.94%	Severe
Slide 13	42,312	97,284	139,596	14,376	153,972	90.66%	Severe
Slide 14	8181	31,021	39,202	10,183	49,385	79.38%	Severe
Slide 15	13,868	41,825	55,693	18,468	74,161	75.10%	Severe
Slide 16	15,857	38,767	54,624	21,462	76,086	71.79%	Severe
Slide 17	43,121	111,433	154,554	82,127	236,681	65.30%	Severe
Slide 18	10,431	25,887	36,318	7456	43,774	82.97%	Severe
Slide 19	14,947	43,166	58,113	21,990	80,103	72.55%	Severe
Slide 20	5392	17,398	22,790	7557	30,347	75.10%	Severe
Slide 21	14,158	36,934	51,092	8229	59,321	86.13%	Severe
Slide 22	5416	17,768	23,184	8314	31,498	73.60%	Severe
Slide 23	4592	13,478	18,070	7838	25,908	69.75%	Severe
Slide 24	7293	21,152	28,445	11,631	40,076	70.98%	Severe
Slide 25	34,745	79,172	113,917	8726	122,643	92.89%	Severe
Slide 26	13,401	36,050	49,451	7341	56,792	87.07%	Severe
Slide 27	8235	25,323	33,558	10,095	43,653	76.87%	Severe
Slide 28	20,544	59,581	80,125	32,069	112,194	71.42%	Severe
Slide 29	9387	26,953	36,340	15,638	51,978	69.91%	Severe
Slide 30	13,542	71,789	85,331	29,630	114,961	74.23%	Severe
Slide 31	24,745	64,878	89,623	29,901	119,524	74.98%	Severe
Slide 32	16,236	44,826	61,062	17,115	78,177	78.11%	Severe
Slide 33	4463	13,836	18,299	2084	20,383	89.78%	Severe
Slide 34	17,357	39,852	57,209	18,079	75,288	75.99%	Severe
Slide 35	5732	9607	15,339	6564	21,903	70.03%	Severe
Slide 36	9369	47,242	56,611	17,007	73,618	76.90%	Severe
Slide 37	17,905	37,926	55,831	31,110	86,941	64.22%	Severe
Slide 38	14,547	40,557	55,104	16,964	72,068	76.46%	Severe
Slide 39	4324	11,052	15,376	5101	20,477	75.09%	Severe
Slide 40	26,063	55,054	81,117	24,916	106,033	76.50%	Severe
Slide 41	7484	16,649	24,133	10,326	34,459	70.03%	Severe
Slide 42	7866	14,009	21,875	13,662	35,537	61.56%	Severe
Slide 43	20,173	55,099	75,272	7534	82,806	90.90%	Severe
Slide 44	4446	34,115	38,561	17,049	55,610	69.34%	Severe
Slide 45	27,344	59,600	86,944	33,972	120,916	71.90%	Severe
Slide 46	11,228	29,264	40,492	10,727	51,219	79.06%	Severe
Slide 47	10,077	15,108	25,185	4165	29,350	85.81%	Severe
Slide 48	8017	22,901	30,918	13,648	44,566	69.38%	Severe
Slide 49	3877	8746	12,623	5948	18,571	67.97%	Severe
Slide 50	3470	11,946	15,416	5835	21,251	72.54%	Severe
Slide 51	4508	12,607	17,115	6261	23,376	73.22%	Severe
Slide 52	2972	6214	9186	4737	13,923	65.98%	Severe
Slide 53	4398	14,407	18,805	8139	26,944	69.79%	Severe
Slide 54	11,109	22,543	33,652	13,157	46,809	71.89%	Severe
Slide 55	3914	7173	11,087	3768	14,855	74.63%	Severe
Slide 56	462	906	1368	510	1878	72.84%	Severe
Slide 57	5614	19,043	24,657	11,951	36,608	67.35%	Severe
Slide 58	8371	16,381	24,752	9971	34,723	71.28%	Severe
Slide 59	3802	16,347	20,149	6059	26,208	76.88%	Severe
Slide 60	7106	17,638	24,744	11,159	35,903	68.92%	Severe
Slide 61	19,229	23,164	42,393	6501	48,894	86.70%	Severe

**Table 7:** Results of nuclei counting and severity level (MYC˗).

MYC˗Slide	Negative Cells	Positive Cells	Abnormal Cells (Negative + Positive)	Normal Cells	Total Number of Cells	Percentage of Abnormal Cells	Severity Level
Slide 1	56,504	8645	65,149	32,641	97,790	66.62%	Severe
Slide 2	35,648	4031	39,679	19,774	59,453	66.74%	Severe
Slide 3	2916	302	3218	2315	5533	58.16%	Severe
Slide 4	2588	199	2787	961	3748	74.36%	Severe
Slide 5	8696	1931	10,627	2007	12,634	84.11%	Severe
Slide 6	3601	956	4557	1415	5972	76.31%	Severe
Slide 7	24,462	5745	30,207	13,455	43,662	69.18%	Severe
Slide 8	797	134	931	118	1049	88.75%	Severe
Slide 9	20,262	8061	28,323	4403	32,726	86.55%	Severe
Slide 10	34,785	6192	40,977	33,168	74,145	55.27%	Severe
Slide 11	32,948	10,217	43,165	22,996	66,161	65.24%	Severe
Slide 12	93,441	17,878	111,319	55,783	167,102	66.62%	Severe
Slide 13	35,909	3699	39,608	17,225	56,833	69.69%	Severe
Slide 14	15,980	4151	20,131	5310	25,441	79.13%	Severe
Slide 15	1021	339	1360	451	1811	75.10%	Severe
Slide 16	3314	1302	4616	1888	6504	70.97%	Severe
Slide 17	64,382	21,119	85,501	33,092	118,593	72.10%	Severe
Slide 18	21,015	4313	25,328	10,204	35,532	71.28%	Severe
Slide 19	51,199	19,372	70,571	24,435	95,006	74.28%	Severe
Slide 20	18,002	5969	23,971	7430	31,401	76.34%	Severe
Slide 21	5881	947	6828	2617	9445	72.29%	Severe
Slide 22	2188	785	2973	906	3879	76.64%	Severe
Slide 23	5789	2511	8300	2828	11,128	74.59%	Severe
Slide 24	15,352	6276	21,628	7459	29,087	74.36%	Severe
Slide 25	7340	562	7902	3467	11,369	69.50%	Severe
Slide 26	2162	321	2483	923	3406	72.90%	Severe
Slide 27	4438	1335	5773	1877	7650	75.46%	Severe
Slide 28	2025	811	2836	978	3814	74.36%	Severe
Slide 29	6019	2591	8610	2999	11,609	74.17%	Severe
Slide 30	5126	1780	6906	1303	8209	84.13%	Severe
Slide 31	24,382	8136	32,518	12,405	44,923	72.39%	Severe
Slide 32	3815	1069	4884	1769	6653	73.41%	Severe
Slide 33	11,538	1313	12,851	4145	16,996	75.61%	Severe
Slide 34	14,633	4623	19,256	8386	27,642	69.66%	Severe
Slide 35	16,317	6983	23,300	13,901	37,201	62.63%	Severe
Slide 36	9236	2774	12,010	2381	14,391	83.45%	Severe
Slide 37	35,403	19,724	55,127	26,021	81,148	67.93%	Severe
Slide 38	83,447	25,691	109,138	39,139	148,277	73.60%	Severe
Slide 39	21,831	7242	29,073	11,372	40,445	71.88%	Severe
Slide 40	35,587	10,932	46,519	22,024	68,543	67.87%	Severe
Slide 41	4086	1748	5834	2622	8456	68.99%	Severe
Slide 42	9026	5636	14,662	8233	22,895	64.04%	Severe
Slide 43	3411	341	3752	1374	5126	73.20%	Severe
Slide 44	436	193	629	82	711	88.47%	Severe
Slide 45	30,762	12,022	42,784	19,626	62,410	68.55%	Severe
Slide 46	26,310	6969	33,279	12,769	46,048	72.27%	Severe
Slide 47	53,547	8855	62,402	41,625	104,027	59.99%	Severe
Slide 48	1183	522	1705	597	2302	74.07%	Severe
Slide 49	23,258	10,960	34,218	15,169	49,387	69.29%	Severe
Slide 50	33,040	12,507	45,547	13,232	58,779	77.49%	Severe
Slide 51	58,803	21,507	80,310	28,718	109,028	73.66%	Severe
Slide 52	8243	4250	12,493	5973	18,466	67.65%	Severe
Slide 53	37,223	16,113	53,336	16,287	69,623	76.61%	Severe
Slide 54	31,589	12,352	43,941	21,653	65,594	66.99%	Severe
Slide 55	43,116	14,657	57,773	31,525	89,298	64.70%	Severe
Slide 56	28,500	10,627	39,127	19,956	59,083	66.22%	Severe
Slide 57	24,878	12,060	36,938	10,893	47,831	77.23%	Severe
Slide 58	54,651	22,020	76,671	39,186	115,857	66.18%	Severe
Slide 59	4762	1432	6194	1441	7635	81.13%	Severe
Slide 60	17,059	7693	24,752	9971	34,723	71.28%	Severe
Slide 61	4328	664	4992	4144	9136	54.64%	Severe

**Table 4:** Accuracies and losses for each epoch with the Adam optimizer (Batch size 64).

Epochs	HoVerNet					VGG16						
	Acc	Loss	Acc	Loss	Acc	Loss	Acc	Loss	Acc	Loss	Acc	Loss
	Training		Validation		Testing		Training		Validation		Testing	
15	100.00	4.45 × 10^−5^	82.99	1.7802	83.43	1.5937	95.79	0.1348	91.95	0.5142	94.81	0.0821
20	100.00	4.54 × 10^−6^	82.96	2.1558	83.87	1.8724	96.67	0.2579	93.63	0.3122	98.63	0.2196
**25**	**100.00**	**2.90 × 10^−7^**	**83.25**	**2.2399**	**84.46**	**2.0036**	**100.00**	**0.0286**	**99.98**	**0.1098**	**99.67**	**0.0112**
30	100.00	7.34 × 10^−8^	82.49	2.8881	83.34	2.5491	99.97	0.2991	99.54	0.5364	98.59	0.2234
35	100.00	6.83 × 10^−7^	79.42	3.9730	79.92	3.6404	97.94	0.3948	96.88	0.4409	96.14	0.1807
45	100.00	1.08 × 10^−7^	80.51	2.9528	81.87	2.7966	96.25	0.7266	93.68	0.7345	94.51	0.3142
50	100.00	4.79 × 10^−7^	81.84	4.0712	82.52	3.7927	96.48	0.7349	98.37	0.9213	99.43	0.2517
100	100.00	5.31 × 10^−5^	74.79	5.0253	74.53	4.7763	-	-	-	-	-	-
800	100.00	1.07 × 10^−7^	74.68	12.4109	75.75	11.9979	-	-	-	-	-	-

Bold: Best accuracy and loss results for HoVerNet and VGG16.

## Data Availability

The original contributions presented in this study are included in the article. Further inquiries can be directed to the corresponding author.

## Abbreviations

The following abbreviations are used in this manuscript:

AI	Artificial Intelligence
CNN	Convolutional Neural Network
DLBCL	Diffuse Large B-Cell Lymphoma
GUI	Graphic User Interface
ROI	Region of Interest
WSI	Whole Slide Image
